# Infectivity of symptomatic *Plasmodium vivax* cases to different generations of wild-caught and laboratory-adapted *Anopheles arabiensis* using a membrane feeding assay, Ethiopia

**DOI:** 10.1016/j.crpvbd.2023.100137

**Published:** 2023-07-27

**Authors:** Tenaye Ayele, Biniam Wondale, Girum Tamiru, Nigatu Eligo, Bernt Lindtjørn, Fekadu Massebo

**Affiliations:** a Department of Biology, Arba Minch University, Arba Minch, Ethiopia; b Department of Biology, Wolaita Sodo University, Sodo, Ethiopia; c Centre for International Health, University of Bergen, Norway

**Keywords:** *Anopheles arabiensis*, *Plasmodoum vivax*, Membrane feeding assays, Ethiopia

## Abstract

When measuring human to mosquito transmission of *Plasmodium* spp., laboratory-adapted (colony) mosquitoes can be utilized. To connect transmission studies to the local epidemiology, it can be important to comprehend the relationship between infectivity in laboratory-adapted (colony) and wild-caught (wild) mosquitoes of the same species. Microscopically confirmed *Plasmodium vivax* cases were recruited from health facilities in Arba Minch town, and a nested polymerase chain reaction (nPCR) was used for subsequent confirmation. We performed paired membrane-feeding assays using colony *An. arabiensis* and three generations of wild origin *An. arabiensis*. *Anopheles arabiensis* aged 3–6 days were fed after being starved for 8–14 h. Microscopically, the oocyst development was evaluated at day 7 after feeding. Circumsporozoite proteins (CSPs) assay was carried out by enzyme-linked immunosorbent assay (ELISA). In 19 paired feeding experiments, the feeding efficiency was more than doubled in colony (median: 62.5%; interquartile range, IQR: 35–78%) than in wild mosquitoes (median: 28.5%; IQR: 17.5–40%; *P* < 0.001). Among the 19 *P. vivax* gametocyte-positive blood samples, 63.2% (*n* = 12) were infective to wild *An. arabiensis* and 73.7% (*n* = 14) were infective to colony *An. arabiensis*. The median infection rate was twice as high (26%) in the colony than in the wild (13%) *An. arabiensis*, although the difference was marginally insignificant (*P* = 0.06). Although the observed difference was not statistically significant (*P* = 0.19), the median number of oocysts per midgut was more than twice as high (17.8/midgut) in colony than in wild (7.2/midgut) *An. arabiensis*. The median feeding efficiency was 26.5% (IQR: 18–37%) in F1, 29.3% (IQR: 28–40%) in F2 and 31.2% (IQR: 30–37%) in F3 generations of wild *An. arabiensis*. Also, no significant difference was observed in oocyst infection rate and load between generations of wild *An. arabiensis*. CSP rate of *P. vivax* was 3.1% (3/97; 95% CI: 0.6–8.8%) in wild and 3.6% (3/84; 95% CI: 0.7–10.1%) in colony *An. arabiensis*. The results of the present study revealed that oocyst infection and load/midgut, and CSP rate were roughly comparable, indicating that colony mosquitoes can be employed for infectivity studies, while larger sample sizes may be necessary in future studies.

## Highlights

• Feeding efficiency was higher in colony compared to wild *Anopheles arabiensis.*
• Median oocyst load/midgut was double in the colony than wild *An. arabiensis*, but not statistically significant.
• Wild *An. arabiensis* feeding efficiency improved over generations but oocyst infection rate did not change significantly.
• CSP infection rate of *Plasmodium vivax* was comparable in wild and colony *An. arabiensis.*
• Colony mosquitoes might be employed for infectivity studies, but research with a larger sample may be needed.

## Graphical abstract

**Figure:**
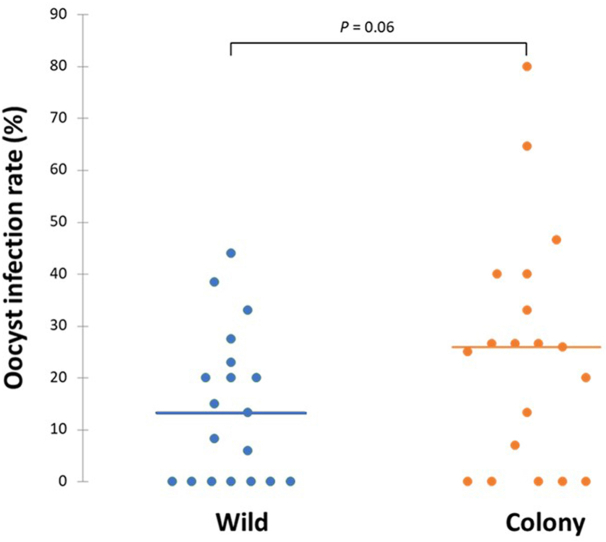

## 1 Introduction

In 2021, malaria continues to be one of the most serious diseases in the globe with 247 million cases and more than 619,000 fatalities, with Africa accounting for 96% of cases and 95% of deaths (WHO, 2022). There are two scenarios in the malaria control and elimination programme; countries with low burdens are making steady progress toward elimination, whereas those with high loads are having challenges (Monroe et al., 2022).

Africa is the heartland of malaria due to the following reasons: the existence of the most effective vectors (*Anopheles gambiae* complex and *Anopheles funestus* group), and the presence of most dangerous parasite species (*Plasmodium falciparum*) that causes most cases and fatalities (Bartilol et al., 2021). Although *Plasmodium vivax* is uncommon in Africa, its prevalence is rising outside of the Horn of Africa (Twohig et al., 2019). Outside of Africa, *P. vivax* and *P. falciparum* parasites coexist, but *P. falciparum* is the main health threat (Hay et al., 2004). In Ethiopia, malaria is widespread, and many people are at risk for this disease. *Plasmodium falciparum* and *P. vivax* are the two species affecting human health (Taffese et al., 2018; Esayas et al., 2020).

There are over 43 species of *Anopheles* mosquitoes in Ethiopia; only a few are either suspected or proven malaria vectors. The primary malaria vector is *Anopheles arabiensis* (Animut et al., 2013; Massebo et al., 2013; Esayas et al., 2020), while *Anopheles pharoensis* plays a secondary role in the transmission of both *P. vivax* and *P. falciparum* parasites (Animut et al., 2013). *Anopheles stephensi* is a newly discovered malaria vector in the east and southeast of the country (Carter et al., 2018; Balkew et al., 2020; Tadesse et al., 2021).

Vector control methods such as indoor residual spraying (IRS) and insecticide-treated nets (ITNs) are the principal tools in the current fight against malaria (Bhatt et al., 2015). However, because of high prevalence of residual malaria transmission, new innovative tools are required to eradicate malaria (Loha et al., 2019; Sherrard-Smith et al., 2019). Understanding the interaction between the *Plasmodium* parasites and the vectors is essential for the development of innovative tools such as transmission blocking vaccines and antimalarial drugs (Ndo et al., 2016; Yu et al., 2022). The specific relationship between the vector and the *Plasmodium* parasite can vary under different conditions, e.g. parasite polymorphisms to evade the mosquito immune system (Molina-Cruz et al., 2017), the mid-gut microbiota (Gomes et al., 2017), larval food and temperature (Lyons et al., 2012). Additionally, the mosquito infection is influenced by the proportion of asexual parasites that develop into gametocytes. As gametocyte density increases, the number of mosquito infections and the parasite load per mosquito increase (Barry et al., 2021).

Numerous studies on *Plasmodium-Anopheles* interactions in malaria endemic areas used populations of colony mosquitoes (e.g. Zhu et al., 2013; Timinao et al., 2021a). Although laboratory-adapted (colony) mosquitoes offer significant advantages over wild mosquitoes in terms of logistics, ease of maintenance, flexibility of scaling-up and reproducibility of experiments (Mohanty et al., 2018), there are a lot of limitations as they are maintained in a regulated environment for long time. Due to inbreeding, genetic drift, and the accumulation of traits that help them survive in artificial breeding conditions, colony mosquitoes may not accurately reflect the genetic make-up of a wild population (Lainhart et al., 2015). The interaction of *Plasmodium* spp. with *Anopheles* spp. in the wild must constantly evolve, and a successful vector-parasite association must depend on the ability of parasites to continuously adapt to the changing ecosystem. However, how mosquito genetic differentiation affects the susceptibility of colonized mosquitoes to *Plasmodium* spp. infection is poorly understood. One way to study the effect of genetic diversity on vector-parasite interactions in wild and colonized mosquitoes would be feeding the same patient-derived *Plasmodium* blood to age-matched wild and laboratory mosquitoes and tracking parasite development with the mosquitoes (Mohanty et al., 2018).

The present study used different generations of wild and colony-maintained *An. arabiensis* in a membrane feeding assay using blood from *P. vivax-*positive patients. The objective of this study was to compare the infectivity of *P. vivax* to different generations of wild and colony *An. arabiensis*, Ethiopia.

## 2 Materials and methods

### 2.1 Description of study setting

Wild mosquito larval sampling was conducted from mosquito breeding sites in Arba Minch and nearby villages, Gamo Zone, southwest Ethiopia. Nearby areas of Arba Minch are characterized by intensive irrigation throughout the year and have permanent water bodies like Lakes Chamo and Abaya and rivers. These water bodies provide favourable breeding sites for mosquitoes at their shores. The climate is hot and humid which is favourable for mosquito breeding and malaria transmission. *Plasmodium falciparum* and *P. vivax* are the two common parasites in the district (Loha et al., 2012). The vectors responsible for malaria transmission are *An. arabiensis* and *An. pharoensis* (Zeru et al., 2020). Malaria patients for this study were recruited from Dil-Fana Primary Hospital and Shecha Health Centre in Arba Minch town.

### 2.2 Study design

**Fig. 1:**
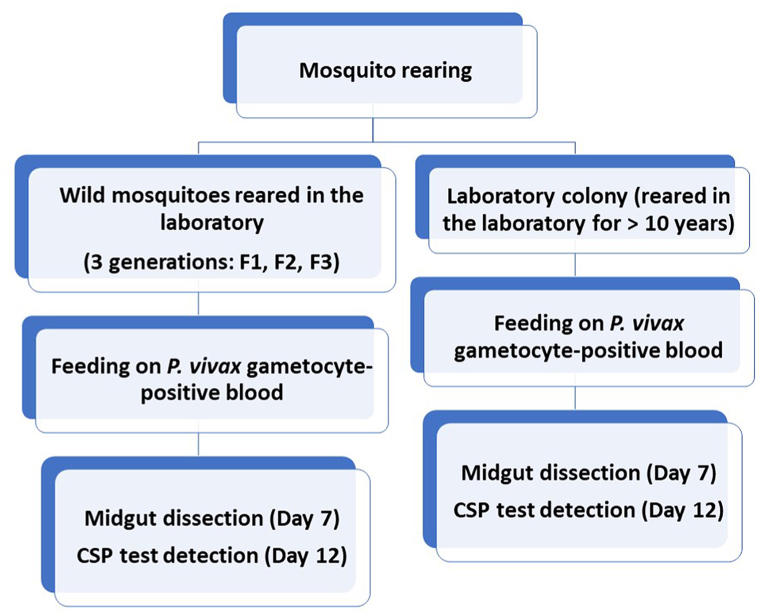
Flow chart of the experimental design.

An open label experimental study design was employed to compare the infectivity of *P. vivax* parasites to different generations of wild-caught (hereinafter referred to as “wild”) and laboratory-adapted (hereinafter referred to as “colony” *An. arabiensis*) (Fig. 1). Mosquitoes aged between 3 and 6 days were exposed to infected blood through an artificial membrane feeding system. Mid-gut dissection was carried out at day 7 post-infection for oocyst detection under light microscopy and circumsporozoite protein (CSP) detection was performed on those mosquitoes that survived until day 12 because of the short sporogonic cycle of *P. vivax* in mosquitoes compared with the sporogonic cycle of *P. falciparum* (Olliaro et al., 2016). Wild (generations F1, F2 and F3) and colony (maintained for more than 10 years in the laboratory) *An. arabiensis* were used for experimental infections.

### 2.3 Sampling and rearing of larvae and pupae of wild *An. arabiensis*

Larvae and pupae were collected from all potential natural mosquito breeding habitats using the standard dipping method. The same habitats were examined repeatedly if they were permanent, such as the shores of lakes and rivers. To avoid larval predators and competitors, the larvae and pupae were promptly filtered using cloth mesh and brought to the Arba Minch University Advanced Medical Entomology and Vector Control Laboratory for rearing. The collected larvae were maintained in plastic trays in the original water collected from the breeding sites and provided with TetraMin fish food (Cichlid Sticks; Tetra, Maidenhead Aquatics, Leicester, UK). Pupae were picked in glass beakers containing tap water and kept in cages until emergence into adults. The emerged adults were maintained in cages on 10% sucrose solution under an optimal temperature of 25–27 °C and a relative humidity of 70–80%. On days 3–6 post-emergence, female mosquitoes were morphologically identified as *Anopheles arabiensis*, the only species of *An. gambiae* complex as confirmed in a previously study (Massebo et al., 2013). Mosquitoes were starved overnight (for 14 h) and then fed on rabbit blood. Fed female mosquitoes were kept inside the cage for oviposition, and their eggs were used for the production of F1 progeny of *An. arabiensis*. Half of the F1 generation of *An. arabiensis* were kept inside the cage for oviposition, and their eggs were developed into F2 progeny. Half of the larvae hatched from these eggs were reared for the experiment while the other half were kept for production of the F2 progeny. Similarly, the F3 generation was maintained for the experiment.

### 2.4 Rearing of colony *An. arabiensis*

*Anopheles arabiensis* colonies that had been maintained for more than 10 years in the Arba Minch University Medical Entomology and Vector Control Laboratory were used for further rearing for the experiments. The adult mosquitoes were fed on rabbit blood, and eggs, larvae and pupae were obtained for further rearing. For wild and laboratory colony development, optimal and comparable laboratory conditions (temperature of 25–27 °C and relative humidity of 70–80%) were maintained. For infection experiments, laboratory-reared 3–6 day-old mosquitoes were used.

### 2.5 Screening patients

Patients who presented to the selected health facilities of Arba Minch town and found to be microscopy-positive with gametocytes of *P. vivax* were asked to donate 3 ml venous blood sample after written informed consent prior to antimalarial treatment. Venous blood (1.5 ml) was added to lithium heparin tubes (BD Vacutainer®) for membrane feeding experiment and 1.5 ml were added to ethylenediamine tetraacetic acid (EDTA) tubes for dried blood spot (DBS) preparation and gametocyte preservation in RNA Protect (Zymo Research, Irvine, CA, USA). Gametocyte densities were quantified on thick blood films against 1000 leukocytes and thin blood films were re-examined at the Advanced Medical Entomology and Vector Control Laboratory to confirm *P. vivax* and exclude others as soon as possible because nPCR was performed at the end of the experiment.

### 2.6 Parasite detection by nested PCR

DNA extraction was performed from the DBS samples on the Whatman filter paper using DNeasy Blood & Tissue Kit (Qiagen, Hilden, Germany). Punch samples made with a 6 mm in diameter hole puncher were added to the tubes. The puncher was sterilized between each interval of a punch sample by dipping it in ethanol and putting it over a Bunsen burner flame. The lysis time was increased to 1 h, and the sample was eluted in 200 μl of elution buffer to collect DNA.

We performed a nested PCR to confirm the identification of *Plasmodium* spp. at genus level and then identified the species following the procedure of Snounou et al. (1993). Samples were probed for *Plasmodium* 18S rRNA to species-specific DNA to confirm the *Plasmodium* species. Amplified samples were electrophoresed on a 2% tris-borate-EDTA (TBE)-agarose gel with GelRed and compared to a 100-bp ladder (Merck, Darmstadt, Germany) to estimate the amplicon size; a sample was considered *P. vivax* if the amplificon size was 120 bp.

### 2.7 Membrane feeding experiments

Depending on the availability of the age-matched mosquitoes, 70–80 wild and 30–50 colonized mosquitoes were used for the membrane feeding experiment. In order to increase the number of fed mosquitoes, we exposed larger numbers of wild than colony *An. arabiensis*, taking into account the low feeding efficiency of the former as reported in another study (Chali et al., 2020). Patient’s venous blood was collected in lithium heparin tubes (Vacutainer, BD) and immediately allowed the mosquitoes to feed on using the membrane feeding system. In each infection experiment, one patient blood sample was utilized to infect both wild and colony mosquitoes at the same time.

Wild *An. arabiensis* were starved for 10–14 h before feeding, as it is considered appropriate for wild-caught mosquitoes to get a sufficient number of fully-fed mosquitoes. Because colony mosquitoes had adapted to an artificial membrane feeding system, they were starving for a shorter period of time (4–6 h) than wild mosquitoes. Feeding was performed in the dark (artificial environment that simulate the night time) for 40–60 min using water-jacketed glass feeders (Coelen Glastechniek, Arnemuiden, The Netherlands) that were covered with an artificial membrane (Parafilm) and connected to a circulating water bath (Julabo) maintained at 37 °C. Feeding efficacy (the proportion of fully-fed mosquitoes) of wild and colony mosquitoes was determined. Unfed and partially fed mosquitoes were removed from the holding cages after examining their abdominal status by a senior entomologist, leaving fully-fed mosquitoes undisturbed. Fully-fed mosquitoes were maintained under the same laboratory conditions using 10% sucrose solution, for oocyst examination and CSP detection. Nineteen paired experiments with independent blood sources were conducted to study the competence of wild *versus* colony mosquitoes to *P. vivax*. Of the 19 paired *P. vivax-*positive blood-feeding assays, 9 were carried out with generation F1, 6 with generation F2, and 4 with generation F3.

### 2.8 Oocyst detection

In each infection experiment, an approximately equal number of wild and colony mosquitoes were dissected under a dissecting microscope using a 40× objective 7 days after feeding, and the oocysts were counted. Mid-guts were removed and stained with 1% mercurochrome solution in phosphate-buffered saline (PBS) (Blagborough et al., 2013). Oocysts were counted under a stereomicroscope. For each infection experiment, the infection rate (No. of infected mosquitoes/Total no. of mosquitoes dissected in individual feeding experiment × 100), and oocyst load (Total no. of oocysts/No. of oocyst-positive mosquitoes in individual feeding experiment) were determined.

### 2.9 Sporoziote detection by ELISA

A proportion of *An. arabiensis* (not dissected for oocyst detection) were kept alive for up to 12 days for circumsporozoite protein (CSP) detection through enzyme-linked immunosorbent assay (ELISA) (Beier et al., 1988). Head and thorax were dissected and digested in grinding buffer using a pestle. Monoclonal antibodies (mAbs) were coated onto the ELISA plate and incubated for 30 min before being aspirated and incubated with blocking buffer for 1 h at room temperature. Samples were incubated for 2 h at room temperature, along with negative and positive controls. Colony mosquitoes were used as a control. Peroxidase conjugate (KPL, Milford, USA) was added to the samples for incubation for 1 h at room temperature. Wells were washed three times with wash solution prior to addition of substrate (SeraCare Life Science, Milford, USA). Samples were incubated for 30 min, before examining color change visually and with iMark Microplate reader (Bio Rad, California, USA) at wavelengths of 405–414 nm.

### 2.10 Statistical analysis

All data were entered and analysed in SPSS version 20 and GraphPad Prism 19 software. Using the median test for independent samples, the feeding efficiency (the proportion of fully-fed mosquitoes) and oocyst load were compared between the wild and colony mosquitoes and the generations of *An. arabiensis* in matched feeding experiments.

## 3 Results

### 3.1 Malariometric information

A total of 20 microscopically confirmed *P. vivax-*positive patients were recruited from Shecha Health Center and Dil-Fana Primary Hospital in Arba Minch town (Table 1). Of these, 19 were *P. vivax* mono-infection and one was negative by nPCR (Table 1). The median age of the patients was 17.5 years, and the majority of patients were males 89.5% (17 out of 19 patients). *Plasmodium vivax*-infected patients had an average asexual parasite density of 7180.2 and a gametocyte density of 342.8.

**Table 1:** Malariometric characteristics of *P. vivax-*infected patients of the Health facilities in Arba Minch town, Ethiopia, included in the study.

Variable	Microscopically confirmed *P. vivax*	PCR-confirmed *P. vivax*
No. of cases recruited	20	19
Sex, % male (*n*/*N*)	90 (18/20)	89.5 (17/19)
Median age, years (interquartile range)	17 (4–31)	16 (4–31)
Asexual parasite density (range)	7180.2 (272–60,000)	–
Gametocyte density (range)	342.8 (120–534)	–

*Abbreviations*: n, number of confirmed cases; N, total number of cases.

### 3.2 Feeding efficiency of wild and colony *An. arabiensis*

**Fig. 2:**
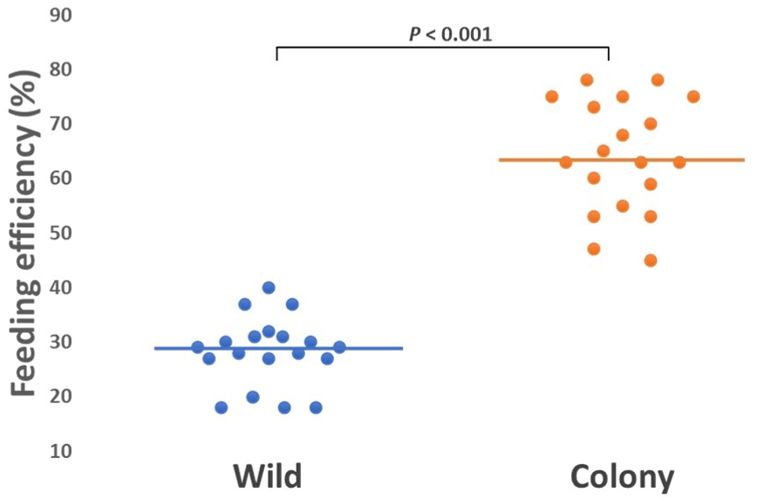
Feeding efficiency (%) of wild and colony *An. arabiensis* using a membrane-feeding system. Dots represent the feeding efficiency of mosquitoes for each *P. vivax-*positive patient blood sample (*n* = 19).

A total of 19 matched membrane feeding experiments with colony and wild mosquitoes were performed on blood samples from *P. vivax-*positive patients. A total of 1508 wild and 754 colony mosquitoes were used for the feeding experiments (Table 2); of these, 423 (28.1%) wild and 481 (63.8%) colony mosquitoes successfully fed a blood meal. The median age of *An. arabiensis* included in the experiments was 5 days (IQR: 3.75–6.00 days, *P* = 0.65). Feeding efficiency varied significantly between wild (median: 28.5%, IQR: 17.5–40%) and colony (median: 62.5%; IQR: 35–78.0%) mosquitoes (*P* ≤ 0.001) (Fig. 2).

**Table 2:** Feeding and infection rate in wild and colony *An. arabiensis* in the membrane-feeding experiments, Ethiopia.

Parameter	Wild	Colony
No. of mosquitoes exposed	1508	754
No. of fed mosquitoes	423	481
Feeding efficiency (% of fully-fed)	28.1	63.8
No. of dissected mosquitoes for oocyst detection	253	256
No. of oocyst-positive mosquitoes	33	69
Oocyst infection rate (%)	13	27
Total oocyst load	475	1242

### 3.3 Feeding efficiency of generations of wild mosquitoes

**Fig. 3:**
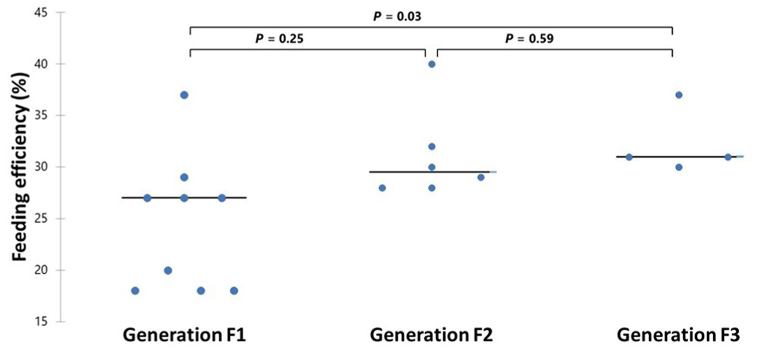
Feeding efficiency (%) of the generations F1–F3 of wild *An. arabiensis* exposed to symptomatic *P. vivax-*positive patient blood using a membrane-feeding system. Dots represent the feeding efficiency of mosquitoes for each *P. vivax-*positive blood sample (*n* = 19).

Three generations of wild *An. arabiensis* were used in feeding experiments. Out of the total exposed mosquitoes, 708 belonged to F1, 480 belonged to F2 and 320 belonged to F3 generation. Of the 19 *P. vivax-*positive blood-feeding assays, 9 (47.4%) were carried out with generation F1, 6 (31.6%) with generation F2, and 4 (21.1%) with generation F3. The median feeding efficiency was 26.5% (IQR: 18–37%) in F1, 29.3% (IQR: 28–40%) in F2 and 31.2% (IQR: 30–37%) in F3 generations of wild *An. arabiensis*. Feeding efficiency differed significantly between F1 and F3 (*P* = 0.03), but there was no difference between F1 and F2 (*P* = 0.25) and F2 and F3 generations (*P* = 0.59) (Fig. 3).

### 3.4 Infection status of wild and colony *An. arabiensis*

**Fig. 4:**
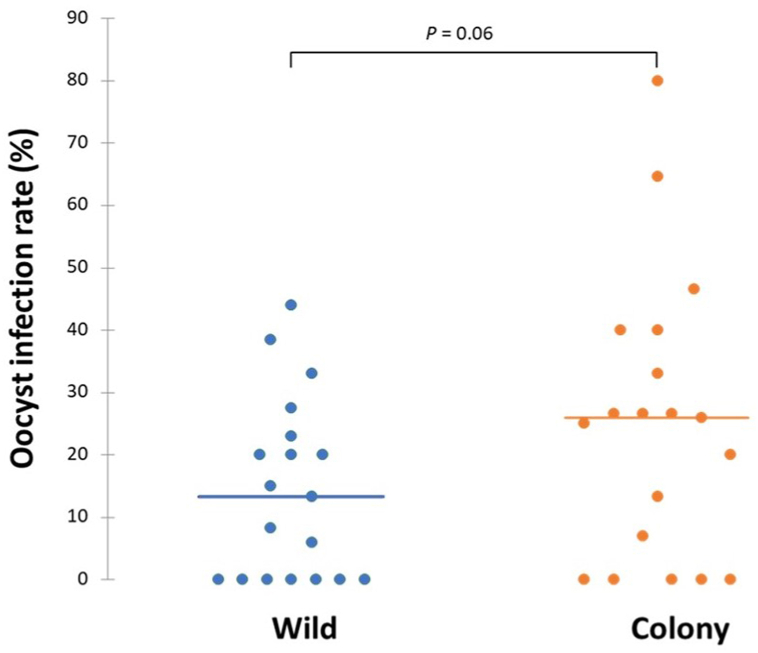
Oocyst infection rate (%) in wild and colony *An. arabiensis* exposed to *P. vivax-*positive patient blood using a membrane-feeding system. Dots represent the infection rate of mosquitoes for each *P. vivax-*positive blood sample (*n* = 19).

Of the 19 *P. vivax* gametocyte-positive blood samples, 63.2% (12/19) were infective to wild *An. arabiensis* and 73.7% (14/19) were infective to colony *An. arabiensis*. In total, 253 wild and 256 colony *An. arabiensis* were dissected in all feeding experiments, with a median oocyst infection rate (prevalence of infection) of 13% (IQR: 0–20.8%) for wild and 26% (IQR: 0–34.8) for colony *An. arabiensis* (Fig. 4). The difference in median infection rate between wild and colony *An. arabiensis* was marginally insignificant (*P* = 0.06).

### 3.5 Oocyst load in wild and colony *An. arabiensis*

**Fig. 5:**
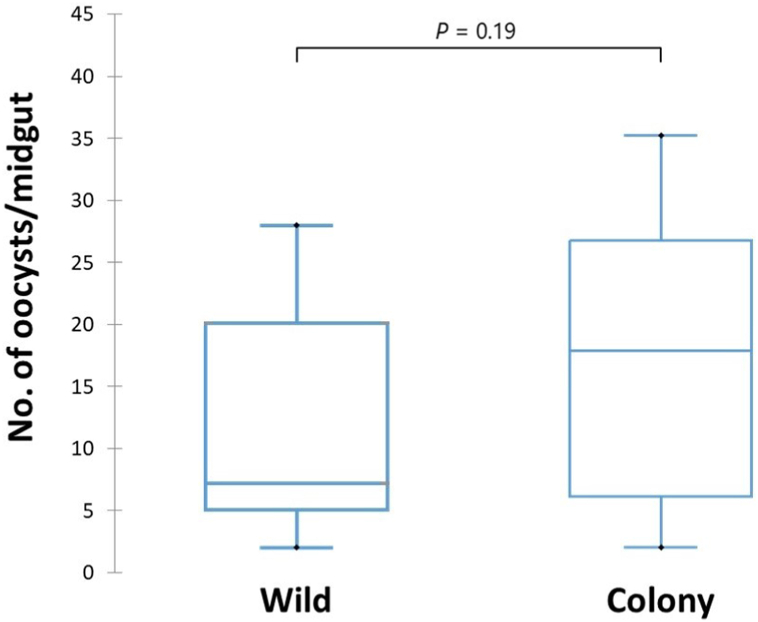
Box-and-whisker plots for oocyst load (no. of oocysts/midgut) in wild and colony *An. arabiensis* exposed to *P. vivax-*positive patient blood using a membrane-feeding system.

A total of 475 oocysts were counted in 33 infected midguts of wild *An. arabiensis* and the overall median oocyst number/infected mosquito midgut was 14.4. A total of 1242 oocyst were counted in 69 infected midguts of colony *An. arabiensis* and the overall median number of oocysts/infected mosquito midgut was 18.0*.* Taking each feeding experiment individually, the number of oocysts observed per midgut ranged from 2 to 28 in wild and from 2 to 38 in colony *An. arabiensis*. The median number of oocyst/midgut was 7.2 in wild *An. arabiensis* and 17.8 in colony *An. arabiensis*, and the observed difference was not statistically significant (*P* = 0.19) (Fig. 5).

### 3.6 Infection rate and oocyst load in F1, F2 and F3 generations of wild *An. arabiensis*

**Fig. 6:**
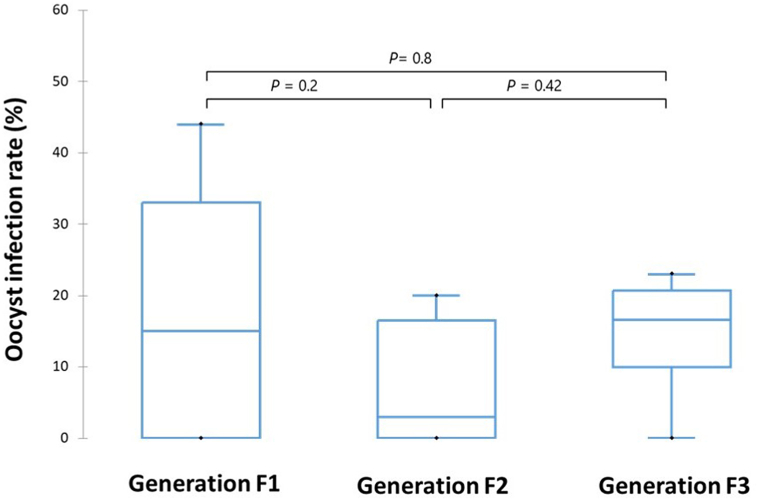
Box-and-whisker plots for oocyst infection rate (%) in three generations (F1, *n* = 9; F2, *n* = 6; F3, *n* = 4) of wild *An. arabiensis* exposed to *P. vivax-*positive blood samples using a membrane-feeding system.

A total of 253 wild *An. arabiensis* were dissected in all 19 feeding experiments. Of these 111 belonged to F1, 84 belonged to F2, and 58 belonged to F3 generation. The median oocyst infection rates were as follows: 15% (range 0–44%) in generation F1; 3% (range 0–20%) in generation F2; and 16.6% (range 0–23%) in generation F3. There was no significant difference in infection rate between generations of wild *An. arabiensis* (Fig. 6).

**Fig. 7:**
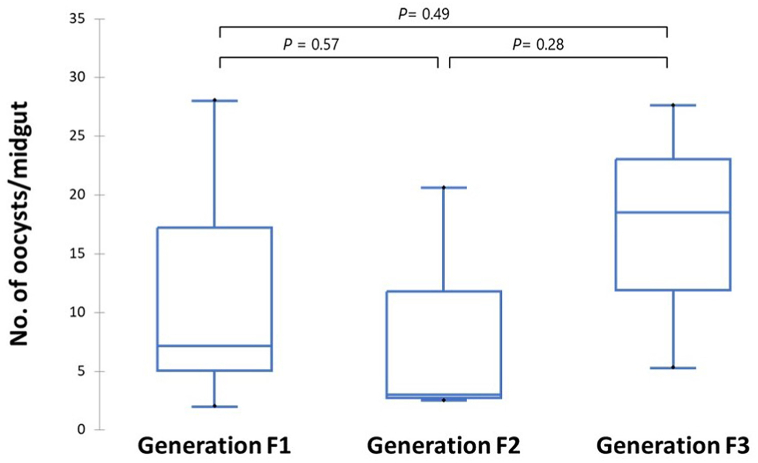
Box-and-whisker plots for oocyst load (no. of oocysts/midgut) in three generations (F1, *n* = 9; F2, *n* = 6; F3, *n* = 4) of wild origin *An. arabiensis* exposed to *P. vivax-*positive blood samples using a membrane-feeding system.

The median number of oocysts/midgut was 7.2 in generation F1, 3.0 in generation F2 and 18.5 in generation F3, and the observed difference was not statistically significant (Fig. 7). Taking each feeding experiment individually, the number of oocyst per midgut ranged as follows: 2.0–28.0 in generation F1, 2.5–20.6 in generation F2, and 5.3–27.6 in generation F3.

### 3.7 Sporozoite infection in wild and colony *An. arabiensis*

On day 12 there were surviving wild and colony *An. arabiensis* in 15 of the 19 feeding experiments; these were used for CSP detection of *P. vivax*. A total of 97 wild and 84 colony *An. arabiensis* were tested for CSP by ELISA. Of these, the *P. vivax* CSP sporozoite rate was 3.1% (3/97; 95% CI: 0.6–8.8%) in the wild and 3.6% (3/84; 0.7–10.1%) in colony *An. arabiensis*.

## 4 Discussion

Feeding efficiency in colony *An. arabiensis* was higher than in the wild *An. arabiensis.* Although the difference was not statistically significant, the oocyst infection rate and oocyst load/midgut were somewhat higher in the colony than in the wild *An. arabiensis*, while the CSP infection rate was essentially comparable; this could imply that colony mosquitoes are less permissive to the development of the infective stage.

Blood-feeding efficiency of colony *An. arabiensis* mosquito was higher than that of wild *An. arabiensis* in line with previous studies (Mohanty et al., 2018; Chali et al., 2020). Optimal numbers of *An. arabiensis* were placed in each cup to improve feeding efficiency, as recommended in a previous study using colony-maintained *An. farauti* (*s.s*.) (Timinao et al., 2021b). Also, wild *An. arabiensis* were starved for an extended time to increase the numbers of fully-fed mosquitoes. Regardless of these, the blood-feeding efficiency of colony *An. arabiensis* was higher than that of wild *An. arabiensis*. The aggression of wild mosquitoes presented difficulties, as evidenced by the nearly 2-fold lower membrane feeding rates for wild than for colony mosquitoes.

Despite the higher infection rate and oocyst load in colony compared with wild *An. arabiensis*, there were no significant variations in infection rate and oocyst load in line with a previous study in Adama, Ethiopia (Chali et al., 2020). The low *Plasmodium* infection rate and oocyst load observed in wild mosquitoes might be explained by physiological susceptibility, as well as variations in the volume and concentration of blood ingested by wild and colony mosquitoes as documented in a previous study (Bousema et al., 2013). The poor feeding success of wild mosquitoes could in turn be due to the change in the emergent environment from the field to the laboratory; mosquitoes being less adapted to the membrane feeding while better adapted to skin feeding (Bousema et al., 2013).

The feeding efficiency was significantly different between generations of wild *An. arabiensis.* Feeding success is gradually improved as the wild mosquito can adapt the laboratory conditions over generations. No difference was observed between generations F1 and F2 of *An. arabiensis*, while the feeding efficiency was significantly higher in F3 than F1. There was no statistically significant difference in infection rate or oocyst load/midgut between the three generations of wild *An. arabiensis*. This could be attributed to the small sample size as well as differences in *P. vivax* gametocyte-positive blood sources (Ndo et al., 2016). We only used three generations of wild *An. arabiensis* for experimental infections, which may not be sufficient to detect expected intergenerational differences. Future studies may test more generations of wild *An. arabiensis* using the same source of parasites to determine intergenerational variation in infectivity.

To minimize the biases regarding the origin of the parasite, wild and colony *An. arabiensis* were exposed to the same blood-meal source in different cups. The study followed standard starvation hours to increase the feeding efficiency and parasite intake. Unlike a comparable study conducted in Ethiopia (Chali et al., 2020), the present study extended the assessment to the sporozoite stage in different generations of wild mosquitoes. The small sample size, which relied on 19 patients albeit utilizing 754 colony and 1508 wild mosquitoes for the feeding efficiency tests, was an important limitation. Hence, it would be debatable whether minor variations would make either wild or colony mosquitoes less suitable for assessing the infectious reservoir in humans or evaluating therapies. Even though the feeding efficiency, infection rate, and the oocyst load was higher in the colony mosquitoes, the sporozoite infection rates were almost identical at the end of the trial. This could imply that colony mosquitoes might be less permissive to sporozoite development than wild mosquitoes, as shown for *An. stephensi* (Mohanty et al., 2018). Except for certain hypotheses, such as genetic alteration during colonization (Lainhart et al., 2015), there is no convincing evidence how the genetic variation is associated with the sporozoite development.

## 5 Conclusions

Although *An. arabiensis* feeding efficiency was higher in the membrane-adapted colony than in the wild mosquitoes, no discernible difference was observed in *P. vivax* oocyst infection rates, oocyst load/midgut and CSP infection rates. Because oocyst infection rate and load/midgut in colony and wild *An. arabiensis* were comparable, colony mosquitoes can be used for infectivity investigations when wild mosquitoes are unavailable. Similar research with a large sample size in diverse contexts with different vector and pathogen species might help our understanding of malaria transmission patterns.

## CRediT authorship contribution statement

**Tenaye Ayele:** Conceptualization, Methodology, Investigation. **Biniam Wondale:** Conceptualization, Methodology, Writing – review & editing, Supervision. **Girum Tamiru:** Methodology, Data curation, Visualization. **Nigatu Eligo:** Methodology, Data curation, Visualization. **Bernt Lindtjørn:** Conceptualization, Validation, Formal analysis, Resources, Writing – review & editing, Supervision, Project administration, Funding acquisition. **Fekadu Massebo:** Conceptualization, Methodology, Validation, Formal analysis, Resources, Writing – original draft, Writing – review & editing, Supervision, Project administration, Funding acquisition.

## Funding

The Norwegian Programme for Capacity Development in Higher Education and Research for Development-Arba Minch University project (ETH-13/0025) financed this study.

## Ethical approval

This study was approved by the Institutional Research Ethics Review Board (IRB) of Arba Minch University (IRB/1150/2021). Written patient consent was obtained before drawing venous blood for membrane feeding. Patients were then immediately treated with current first-line antimalarial drugs according to Ethiopian national malaria treatment guidelines (MOH, 2018).

## Declaration of competing interests

The authors declare that they have no known competing financial interests or personal relationships that could have appeared to influence the work reported in this paper.

## Data Availability

Data supporting the conclusions of this article are provided within the article. Raw data will be made available upon request.
